# Generative AI cybersecurity and resilience

**DOI:** 10.3389/frai.2025.1568360

**Published:** 2025-06-02

**Authors:** Petar Radanliev, Omar Santos, Uchenna Daniel Ani

**Affiliations:** ^1^Department of Computer Sciences, University of Oxford, Oxford, United Kingdom; ^2^Alan Turing Institute, British Library, London, United Kingdom; ^3^Cisco Systems, RTP, San Jose, NC, United States; ^4^School of Computer Science and Mathematics, Keele University, Keele, United Kingdom

**Keywords:** generative artificial intelligence, shadow AI, policy development, responsible AI deployment, data ethics, cybersecurity

## Abstract

Generative Artificial Intelligence marks a critical inflection point in the evolution of machine learning systems, enabling the autonomous synthesis of content across text, image, audio, and biomedical domains. While these capabilities are advancing at pace, their deployment raises profound ethical, security, and privacy concerns that remain inadequately addressed by existing governance mechanisms. This study undertakes a systematic inquiry into these challenges, combining a PRISMA-guided literature review with thematic and quantitative analyses to interrogate the socio-technical implications of generative Artificial Intelligence. The article develops an integrated theoretical framework, grounded in established models of technology adoption, cybersecurity resilience, and normative governance. Structured across five lifecycle stages (design, implementation, monitoring, compliance, and feedback) the framework offers a practical schema for evaluating and guiding responsible AI deployment. The analysis reveals a disconnection between the fast adoption of generative systems and the maturity of institutional safeguards, resulting with new risks from the shadow Artificial Intelligence, and underscoring the need for adaptive, sector-specific governance. This study offers a coherent pathway towards ethically aligned and secure application of Artificial Intelligence in national critical infrastructure.

## 1 Introduction

Artificial Intelligence (AI) operates through self-evolving uses that can autonomously produce new data outputs. Generative AI represents a significant departure from classical algorithmic methods. Generative AI use advanced deep learning frameworks such as Generative Adversarial Networks (GANs) (Goodfellow et al., 2014) and Variational Autoencoders (VAEs). These architectures facilitate the generation of high-dimensional data by employing latent space manipulation and probabilistic modelling. GANs, for instance, employ a dual-network approach, consisting of a generator and discriminator, engaged in a zero-sum game to improve output quality iteratively. In parallel, VAEs focus on encoding data distributions into lower-dimensional latent spaces, from which new samples can be generated. These models are not confined to traditional data outputs. Still, they can instead synthesize intrinsically new outputs, ranging from high-resolution images to contextually rich natural language sequences, often indistinguishable from human-created content.

Generative AI has been deployed in several sectors, each using its unique capacity for autonomous creation. In the creative industries, the automation of content generation (be it in visual art, music composition, or text production) challenges the very notion of human creativity and authorship. Within biomedicine, generative models are accelerating drug discovery by designing novel molecular structures and improving diagnostic accuracy through synthetic medical imaging. Cybersecurity applications exploit generative AI for automated threat detection and adversarial attack simulation, enhancing defensive strategies and offensive capabilities.

However, the increasing reliance on generative AI introduces many challenges. Ethical concerns are at the top, particularly in deepfakes and algorithmic bias. Deepfake technologies, driven by GANs, have shown an unsettling ability to create hyper-realistic yet entirely fabricated audio-visual content, posing risks to information integrity and public trust. Meanwhile, the unintentional propagation of biases embedded in training data can lead to discriminatory outcomes in decision-making systems, exacerbating social inequities.

From a security perspective, generative AI introduces potential attack vectors. Its capability to autonomously generate code or craft sophisticated phishing schemes increases the scale and complexity of cyber-attacks. These threats are intensified by using generative AI to automate misinformation campaigns, where false narratives can be rapidly disseminated, further complicating detection and mitigation efforts.

Privacy concerns also take center stage, particularly regarding the use of personal data in training these expansive models. The vast datasets required to fine-tune generative architectures often include sensitive information, raising profound questions about data ownership, consent, and the potential for re-identification in anonymized datasets. These evolving technologies continually test the legal and regulatory frameworks governing AI applications, including the General Data Protection Regulation (GDPR) (GDPR, 2018; ICO, 2018), necessitating more robust and contextually adaptive governance.

### 1.1 Resilience in generative AI cybersecurity

Resilience in complex systems refers to the ability to anticipate, absorb, recover from, and adapt to adverse conditions. In the context of generative AI, resilience must be evaluated through its capacity to withstand cyber threats, mitigate risks, and ensure robust governance mechanisms that preserve societal stability. We need new governance frameworks for enhancing resilience by establishing risk mitigation strategies that address AI-generated threats while promoting a sustainable and adaptive regulatory environment.

From a cybersecurity perspective, resilience is traditionally assessed by analyzing how a system functions under stress. Generative AI introduces novel risks, such as adversarial attacks, automated misinformation propagation, and large-scale privacy breaches, which can compromise the integrity of digital ecosystems. We need new frameworks that quantifies these risks by measuring the impact of generative AI in adversarial scenarios, ensuring that security vulnerabilities do not erode trust in AI-driven infrastructures.

In this paper, risk assessment for the shadow AI serves as a mechanism for evaluating the resilience of generative AI. We measure resilience by examining how AI systems respond to adversarial shocks, such as:

- *Data Poisoning and Model Robustness:* The resilience of generative AI models depends on their ability to maintain integrity when exposed to manipulated training datasets. Our framework incorporates adversarial training and differential privacy techniques to fortify models against such attacks.
- *Deepfake and Misinformation Detection:* The proliferation of deepfake technology presents significant societal risks. Our framework enhances resilience by integrating AI-driven detection mechanisms to counteract misinformation and preserve digital authenticity.
- *Governance and Policy Enforcement:* Regulatory oversight is essential for resilient AI ecosystems. By embedding security compliance and ethical AI governance, our framework ensures that generative AI operates within well-defined constraints, enhancing its adaptability and sustainability in dynamic threat landscapes.

### 1.2 Research gap

While the current body of research has been predominantly centered on advancing the technical capabilities of generative AI, there remains a deficiency in examining the broader ethical (Jobin et al., 2019; European Commission, 2018; IEEE, 2023; Roberts et al., 2021; Mökander et al., 2024), security (He et al., 2024; Porambage et al., 2019; Sarker et al., 2021; Mishra, 2023; Deng et al., 2024), and privacy (Bartoletti, 2019) implications accompanying its widespread deployment (Jobin et al., 2019; European Commission, 2018; IEEE, 2023). Existing scholarship has largely prioritized algorithmic efficiency and model performance improvements, often neglecting the complex socio-technical ramifications of integrating these systems into various sectors. This oversight is particularly problematic given the rapid pace of generative AI’s advancement, which outstrips the development of corresponding governance frameworks, ethical guidelines, and security protocols (Tedeneke, 2023; Kendzierskyj et al., 2024).

The divided nature of scholarly discourse compounds this issue. Research is siloed into specialized domains without a holistic approach that addresses the intersectionality of ethical, security, and privacy concerns. Ethical challenges, such as algorithmic bias and the generation of misleading content (Orphanou et al., 2022), are often discussed in isolation from security vulnerabilities (CVE, 2022; Miaoui and Boudriga, 2019), such as adversarial attacks (Sun et al., 2018; Carlini and Wagner, 2017; Ren et al., 2020; Chen et al., 2020; Sava et al., 2024; Sava et al., 2022; Wang et al., 2024; Du et al., 2024; Zbrzezny and Grzybowski, 2023; Khamaiseh et al., 2022), and privacy breaches, like the unauthorized exploitation of personal data (Esteve, 2017; Guy and PAlex, 2015; Wheatley et al., 2016; African Union, 2020). This lack of integration results in an incomplete understanding of the full spectrum of risks posed by generative AI technologies.

Moreover, discussions regarding the responsible application of generative AI are still in their infancy. While some initial steps have been made towards establishing regulatory frameworks, many remain embryonic and lack the robustness to manage the multifaceted risks inherent in this rapidly advancing field. The absence of comprehensive, context-specific guidelines further exacerbates the potential for misuse, leaving a critical gap in the literature that necessitates immediate scholarly attention. This gap represents an urgent opportunity for academic contributions that bridge theoretical exploration and provide practical frameworks for generative AI systems’ ethical, secure, and private deployment.

### 1.3 Objectives and contributions

**Figure 1:**
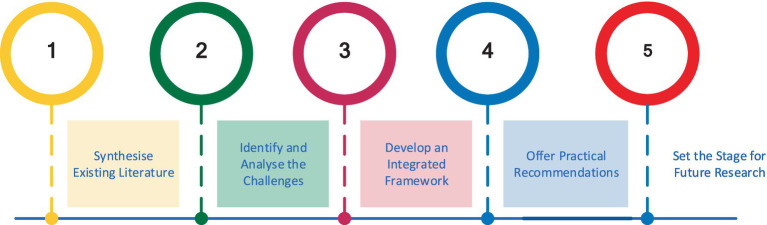
Key objectives.

The principal objective of this paper is to construct a comprehensive and integrated framework that captures the ethical, security, and privacy dimensions of generative AI while concurrently advocating for fostering technological innovation. This framework seeks to balance the requirement of advancing AI capabilities and mitigating associated risks (see key objectives in Figure 1), ensuring that the deployment of generative AI adheres to responsible standards.

To achieve this, the paper will address the following key objectives:

1. This paper synthesis the current research landscape, consolidating unrelated strands of discourse surrounding generative AI. It also critically examines technological advancements and emergent ethical, security, and privacy challenges in deploying generative AI.
2. Analysis of the specific risks and challenges posed by generative AI, with a particular focus on the ethical dilemmas (e.g., bias propagation, misinformation), security threats (e.g., adversarial attacks, automation of cyber threats), and privacy infringements (e.g., re-identification risks in anonymized data).
3. Proposes a multi-layered framework that provides a unified structure for addressing these ethical, security, and privacy challenges. This framework offers a practical utility to various stakeholders, including AI practitioners, policymakers, and regulatory bodies, to guide the responsible deployment of generative AI technologies.
4. Formulates actionable guidelines to ensure that generative AI systems are developed and deployed in accordance with ethical principles, robust security measures, and privacy protections. These recommendations are tailored to the needs of various stakeholders, including developers, users, and regulators.
5. Identify key gaps in the existing literature and propose directions for future research. This includes suggestions for interdisciplinary collaboration to explore the evolving challenges associated with generative AI and its responsible governance.

## 2 Research methodology

This study employs a mixed-methods research design, integrating quantitative and qualitative approaches to capture generative AI’s complex and multi-dimensional nature. This methodological framework is selected to provide a comprehensive analysis of the economic, ethical, and technological aspects of generative AI, which are inherently interconnected but often studied in isolation. Combining empirical data and expert insight ensures that the research addresses measurable outcomes and the more subtle, qualitative dimensions of AI’s broader societal implications.

### 2.1 Quantitative analysis

The quantitative component of the study focuses on a statistical examination of generative AI’s impact across various sectors. Market trends, economic repercussions, and technological advancements are analyzed to quantify the scope and trajectory of generative AI integration into healthcare, cybersecurity, and creative industries. Secondary data sources, including market reports, publicly available databases [e.g., from the International Data Corporation (IDC) and the Institute of Electrical and Electronics Engineers (IEEE)], and industry publications, are leveraged for this analysis.

Analytical techniques employed in the quantitative phase include:

- Regression analysis to assess relationships between the adoption of generative AI and its economic impact across different industries.
- Time-series analysis to track the evolution of generative AI technologies and market responses over time.
- Predictive modelling to forecast future developments and potential disruptions brought about by generative AI in various sectors.

These techniques are facilitated through statistical tools such as SPSS and data analysis libraries in Python (e.g., Pandas and NumPy), ensuring a robust and data-driven analysis of generative AI’s economic and technological footprint.

### 2.2 Qualitative analysis

The qualitative component is centered on the thematic analysis of scholarly literature, expert interviews, and white papers. This methodology aspect is critical for capturing the nuanced ethical, security, and privacy implications of generative AI—issues that are often difficult to quantify but essential to responsible deployment.

Primary qualitative data sources include:

- In-depth interviews with industry experts and academic specialists in AI, focusing on their perspectives regarding the ethical challenges, security vulnerabilities, and privacy concerns related to generative AI technologies.
- A comprehensive review of peer-reviewed academic articles, industry white papers, and regulatory documents to establish the current state of discourse surrounding the responsible implementation of generative AI.

Thematic analysis is conducted using NVivo software, allowing for the systematic coding of qualitative data to identify recurrent themes, patterns, and emergent insights. This method provides an analytical framework to explore areas that quantitative data alone may not reveal, such as the potential for generative AI to exacerbate biases or be exploited in malicious cyber-attacks.

### 2.3 Data integration and analysis

By integrating quantitative metrics and qualitative insights, the study adopts a holistic approach that ensures the validity and reliability of the findings. Quantitative results provide a broad, empirical understanding of generative AI’s economic and technological impact, while qualitative insights offer depth and context regarding the ethical, security, and privacy challenges. This dual approach allows the research to align closely with the study’s objectives and maintains empirical rigor and contextual relevance.

Quantitative data is primarily obtained from market reports, industry analyses, and academic publications. Qualitative data is sourced through expert interviews and a review of pertinent literature. This blend of data ensures the research captures the breadth and depth of generative AI’s implications.

### 2.4 Analytical techniques

The following analytical techniques are employed to ensure rigor:

- Regression and predictive modelling to forecast the future trajectory of generative AI’s influence across industries.
- Time-series analysis to assess the evolution of generative AI applications and their implications over time.
- Thematic coding for identifying and analyzing patterns in expert interviews and literature on the ethical, security, and privacy concerns surrounding generative AI.

Combined with SPSS and Python libraries, these tools ensure a methodologically sound, data-driven analysis that aligns with the study’s objectives.

This comprehensive methodological approach ensures that the study addresses the multi-faceted nature of generative AI, providing a rigorous foundation for the research findings and allowing for the synthesis of empirical evidence and contextual insight. By doing so, the methodology aligns with the study’s overarching aim to deliver a balanced, well-supported framework for understanding and addressing the implications of generative AI.

## 3 Literature review and bibliometric analysis—with visual examples

The literature review and bibliometric analysis are conducted throughout the research article and are addressing specific aspects of the study. This research methodology was chosen to ensure specific sections are developed with references to relevant literature on the specific issues addressed in specific sections of the article. The brief review below provides an examination of generative AI technologies, their practical applications, and their various security, ethics, and privacy challenges.

### 3.1 Theoretical background of generative AI technologies

Generative Adversarial Networks (GANs) (Goodfellow et al., 2014) and Variational Autoencoders (VAEs) (Kutuzova et al., 2021; Da Silva-Filarder et al., 2021; Lawry Aguila et al., 2023; Shi et al., 2019) are two facets of generative AI that have transformed the field of image synthesis and medical imaging, respectively (Kenfack et al., 2021; Ding et al., 2021; Antoniou et al., 2018; Wang et al., 2023; Radford et al., 2015; Wang et al., 2022; Karras et al., 2019; Buzuti and Thomaz, 2023; Beers et al., 2018; Dar et al., 2019; Sandfort et al., 2019; Sindhura et al., 2022; Welander et al., 2018). GANs have the potential to generate hyper-realistic images, as demonstrated by StyleGAN (Karras et al., 2019) in creating highly realistic human faces. GANs have expedited drug discovery processes in the pharmaceutical industry, as shown by Zhavoronkov et al. (2019), where novel molecules were designed in a notably short time frame.

VAEs, on the other hand, have notably impacted medical imaging by enhancing MRI accuracy (Hosny et al., 2018), thereby improving diagnostic methodologies. This is indicative of the broad scope of generative AI technologies.

Transformer-based models, such as GPT-3 (Brown et al., 2020), further expand the application horizon of generative AI. These models can generate text indistinguishable from human writing, which has significant implications across sectors such as journalism and creative industries. This underlines the versatile applications of generative AI.

Generative AI is a type of AI model that can create new data samples that resemble a given input data set. It differs from discriminative models that classify or differentiate between data points. Generative models can be used in various media, such as text, images, video, and audio. For example, they can generate coherent paragraphs for automated storytelling or news article generation, produce new images that were not part of the original dataset, create new video sequences or modify the existing ones for video editing and movie production. They can also produce sound or modify existing audio tracks for music composition and voice generation.

There are several real-world examples of generative AI, such as wearable sensors in healthcare that detect irregular heart rhythms and conduct ECGs; generative AI in art, where artists use GANs to create visual art pieces; accelerometer datasets for fitness apps that track and analyze physical activity; and generative AI in video games, which uniquely generates planets, species, and terrain for the game.

### 3.2 Why the hype around generative AI?

Generative AI has exploded with significant implications for technology, economics, and society. From generating hyper-realistic images to creating new kinds of music, this technology fundamentally reshapes how we create and consume content.

**Figure 2:**
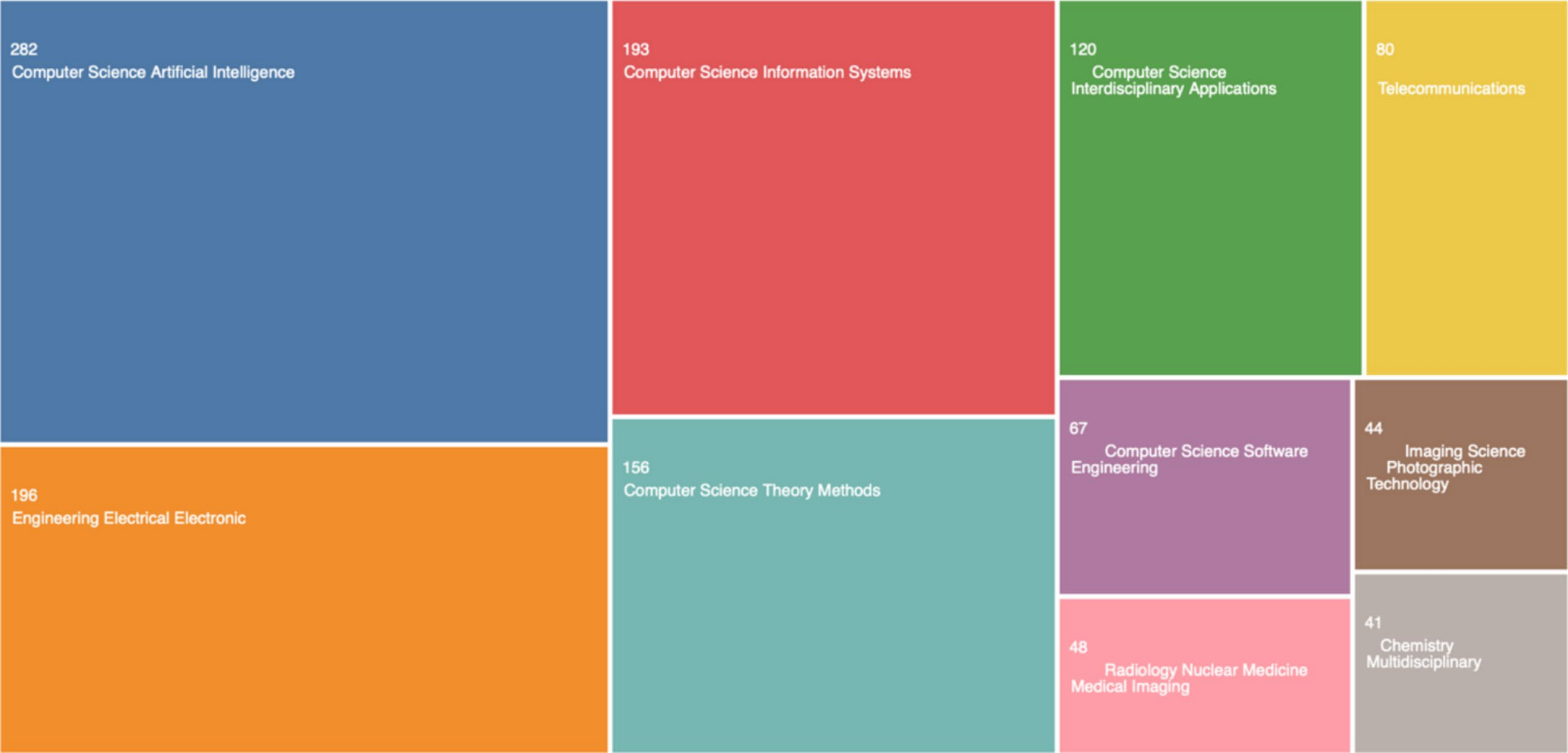
Search results on “generative AI” from the Web of Science core collection.

A generic search on the Web of Science Core Collection for “Generative AI” (as of September 4, 2023) returns only 1,195 publications (see breakdown in Figure 2).

**Figure 3:**
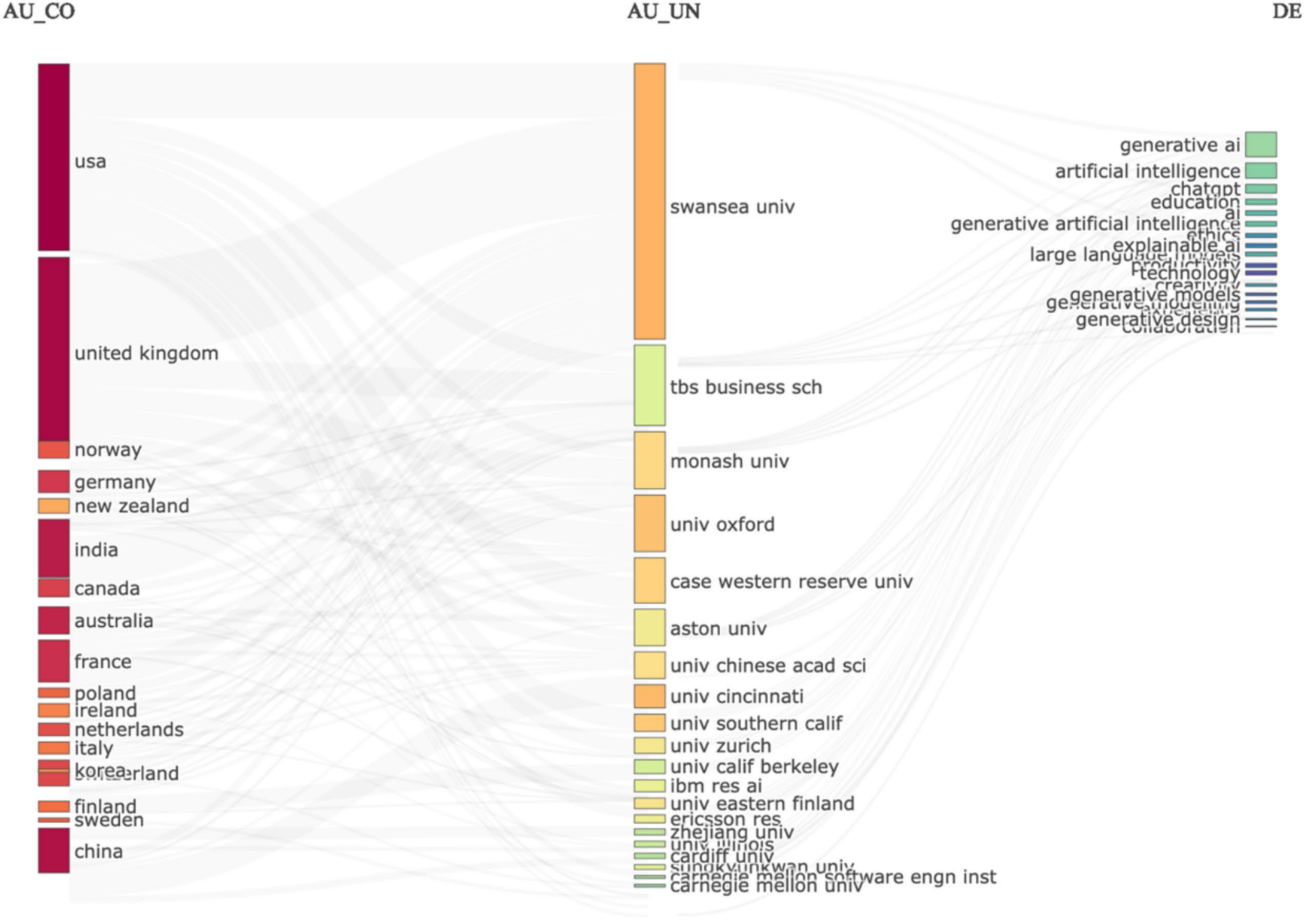
Three fields plot.

We extracted the data records as a file and analyzed them with R to extract further input from the data. In Figure 3, we created a three-field data plot to compare output by country, institution, and keywords. The data analysis results are somewhat unconvincing because, despite all recent developments in the United States, the three-field plot in Figure 3 shows that Swansea University is leading in research output on Generative AI. This shows an error in the data set, or an error in the analysis of the data set, and requires further analysis. For clarity, and for reproducing the same results, we share the data set with other researchers to analyze and identify the causes of this result, but for the purpose of this study, we chose to analyze further data sets, and apply different methods of analysis.

**Figure 4:**
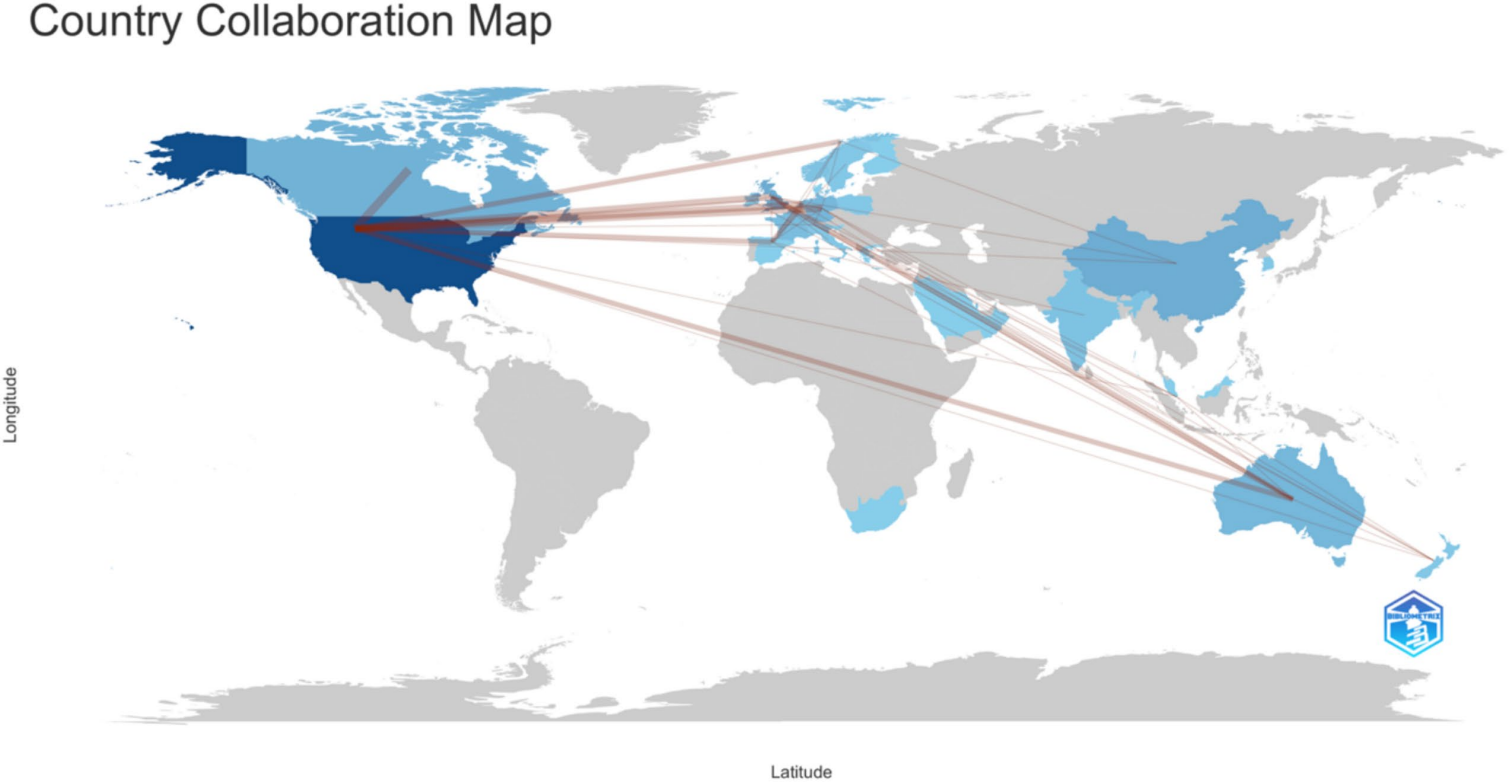
Social structure.

Given that Figure 2 results are unconvincing, we continued analyzing this data file with various statistical approaches. We derived a very different visualization of collaborations in the data: the social structure of the data is analyzed as a country collaboration world map (Figure 4).

**Figure 5:**
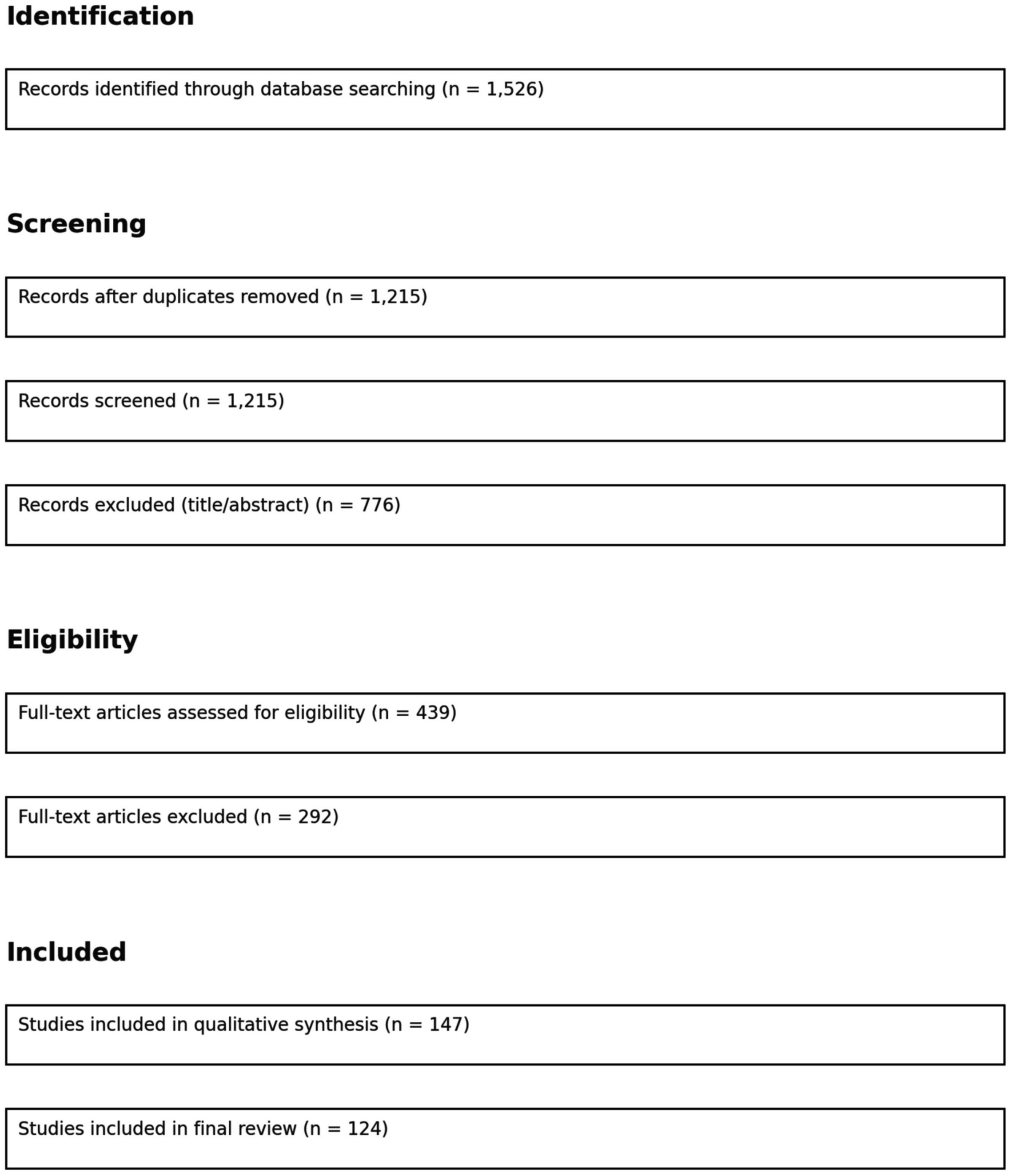
PRISMA flow diagram for the systematic literature review.

The results show in Figure 4, clearly show that the social structure of research output on this topic is strongly corelated to the US. This is significantly different than the results in Figure 3, and yet, its different analysis of the exact same dataset from the Web of Science Core Collection. This clearly descries why simply taking data records from the Web of Science Core Collection, Scopus, or any other database, without applying a strong research methodology, can lead to bias and errors in the data analysis. The next section (Figure 5) details the structured review approach that was selected for eliminating these errors in the datasets and the data analysis process. The two figures (Figures 3, 4) are included for illustrative purposes only, to justify the need for a strong research methodology, which is detailed in the following section.

## 4 Literature review methodology: a PRISMA-guided approach

To ensure methodological rigor and transparency, we conducted a systematic literature review following the PRISMA (Preferred Reporting Items for Systematic Reviews and Meta-Analyses) framework. This approach allowed us to comprehensively identify, select, and synthesize relevant academic and grey literature on the ethical, security, and privacy implications of generative AI.

### 4.1 Identification

We initiated a comprehensive search across four major academic databases: Web of Science, Scopus, IEEE Xplore, and ACM Digital Library. The following Boolean keyword strategy was employed:

(“Generative AI” OR “Generative Adversarial Networks” OR “VAEs” OR “Transformer Models”) AND (“Security” OR “Privacy” OR “Ethics” OR “Governance” OR “Resilience”).

The search was limited to peer-reviewed journal articles and conference papers published between January 2019 and September 2024 to ensure a focus on recent and high-impact literature. We also screened reputable white papers from institutions such as the IEEE, NIST, and OECD.

This process yielded 1,526 unique records.

### 4.2 Screening

All search results were exported to Zotero for reference management. After automatic and manual removal of duplicate entries (*n* = 311), the remaining 1,215 studies underwent a title and abstract screening. Two independent reviewers assessed the relevance based on predefined inclusion and exclusion criteria (see below).

- Inclusion criteria: Studies focused explicitly on generative AI and its cybersecurity, ethical, or privacy implications; articles proposing frameworks, empirical results, or taxonomies.
- Exclusion criteria: Editorials, news articles, opinion pieces, papers focused solely on model architecture without application discussion.

Following this screening phase, 439 papers were selected for full-text analysis.

### 4.3 Eligibility

Full texts of the remaining articles were reviewed to assess methodological soundness and thematic alignment. Papers that lacked sufficient empirical basis or did not engage with the socio-technical aspects of generative AI were excluded. A final set of 147 articles were deemed eligible.

### 4.4 Inclusion

Of the eligible articles, we included 112 peer-reviewed articles and 12 white papers in the final synthesis. These sources were coded using NVivo to identify thematic clusters around ethical governance, adversarial robustness, privacy preservation, and regulatory gaps.

The final selection of studies, as illustrated in Figure 5, provides a robust foundation for understanding the multi-dimensional risks and governance challenges associated with generative AI. By employing NVivo to thematically code the included literature, we identified recurring patterns and conceptual gaps across four primary domains: ethical governance (e.g., fairness, accountability), adversarial robustness (e.g., attack surface analysis, model poisoning), privacy preservation (e.g., data minimization, anonymization), and regulatory frameworks (e.g., GDPR compliance, sector-specific guidelines). This structured analysis ensured methodological transparency and facilitated the development of an integrated framework that synthesizes technical, ethical, and policy-driven insights. The resulting evidence base serves as a critical scaffold for the subsequent theoretical and empirical components of this study.

### 4.5 Generative AI in real-world use cases: review of case study examples from healthcare and climate data analysis

**Figure 6:**
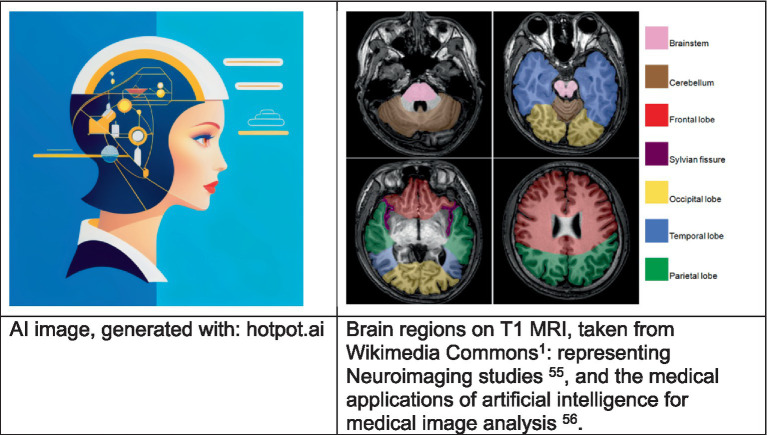
Generative AI enables synthetically generated MRI and X-rays that represent specific diseases and illnesses (Gifford et al., 2020; Sollee et al., 2022).

Generative AI has exemplified the development of dynamically generated video game environments that adapt to individual playstyles. In the medical field, synthetic data creation for training algorithms stands out, offering enhanced diagnostic capabilities while safeguarding patient privacy. These developments, previously envisaged as distant possibilities, are now tangible realities, owing to the transformative impact of generative AI. Imagine video games with worlds generated on the fly, adapting to your playstyle. This is close in reality. In Figure 6, we can see a visual demonstration of an image generated on the fly, and the potential for such image generations is unlimited, even with the current technologies. Consider synthetic data that can train medical algorithms (e.g., MRI, X-Rays), improving diagnostics without compromising patient privacy. Although the image in Figure 6 seems far-fetched in comparison to a medical image, this is just a demonstration of what generative AI is capable of, in other research projects, we use advanced and synthetically generated MRI and X-rays that are representative of specific diseases and illnesses, and we train the AI to detect specific conditions, and this is happening now. Generative AI enables technological leaps we could not have imagined a decade ago.

However, the rise of generative AI is accompanied by a complex array of ethical considerations that require analysis from different perspectives. The potential for ingrained biases and a lack of impartiality within AI systems is a real concern.

Another critical dimension concerns accountability and transparency in AI decision-making processes. Buolamwini and Gebru’s 2018 research (Buolamwini and Gebru, 2018) sheds light on profound racial and gender biases in facial recognition technologies. These findings challenge the prevailing assumptions about the responsibility and openness of AI systems.

Furthermore, AI’s broader societal and employment implications represent a primary area of concern. Acemoglu and Restrepo’s 2020 discourse (Acemoglu and Restrepo, 2019) expanded into AI’s broader social repercussions, particularly focusing on its effects on employment patterns and economic disparities. These considerations underscore the need for a balanced approach to harnessing the potential of generative AI while mitigating its unintended consequences.

### 4.6 Societal impact

**Figure 7:**
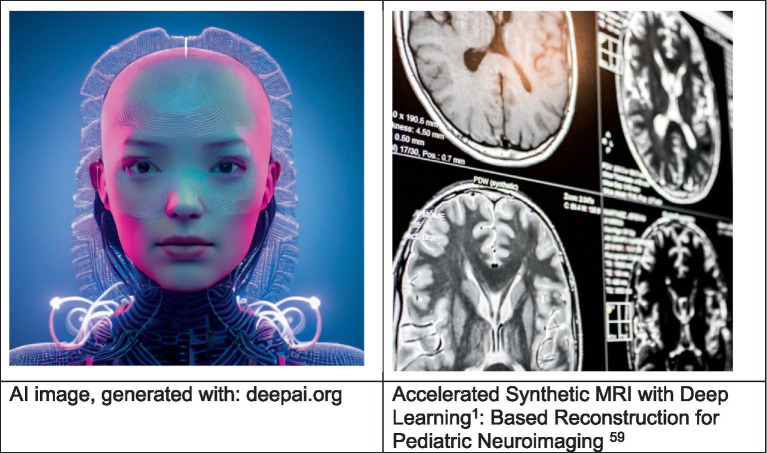
Generative AI democratizes creativity, and synthetic images are valuable in medical applications (Kim et al., 2022).

Generative AI extends beyond technological and business applications, indicating an era where creativity is democratized. This innovation enables those without artistic backgrounds to produce artistic imagery through AI tools. In healthcare, the advent of personalized treatments tailored to individual health profiles is now a growing possibility. We stand at the cusp of an era where personal experiences can be profoundly customized through these generative models. However, this progress brings significant privacy concerns. Generative AI democratizes creativity, and synthetic images are valuable in medical applications. For example, a medical practitioner without artistic skills and capabilities can create compelling visuals using AI tools and images (see Figure 7). Even if such images are not of the same quality and creativity as real artists, the images can be developed according to what the medical practitioners require and what the AI system needs to be trained. Such images would enable medical practitioners to visualize the body’s composition without intrusive procedures. In medicine, personalized treatments could be generated based on individual health data. Our personal experiences and professional requirements can be deeply customized with generative AI models.

The issue of Consent and Anonymization is critical, as demonstrated by Rocher et al. (2019). Their research revealed the startling ease with which supposedly anonymised data could be re-identified, underscoring the urgent need for robust data protection measures.

The Cambridge Analytical scandal, reported by Cadwalladr and Graham-Harrison in 2018 (Carole and Emma, 2018), starkly illustrates the risk of data misuse. This incident serves as a stark reminder of the dangers inherent in the mishandling of personal data and highlights the necessity for ethical data management practices. As Kostka discusses (Kostka, 2019), AI has amplified concerns about surveillance and monitoring in systems such as China’s social credit scheme (Kostka, 2019). The application of AI in these surveillance contexts raises significant privacy issues, necessitating a balanced approach to deploying AI technologies.

### 4.7 Economic considerations

The economic landscape of generative AI is set for considerable growth, reflecting its transformative potential across various industries. Although precise predictions for the market size vary, the trajectory suggests a significant financial impact. Generative AI is expected to significantly contribute to the broader AI market, which is experiencing rapid expansion.

The cost-efficiency aspect of generative AI is particularly noteworthy. Using synthetic data to train models can reduce data collection and processing expenses. This cost-saving factor is financially advantageous and contributes to accelerated development cycles for AI models, enabling swifter deployment and realizing technological benefits.

Moreover, generative AI is anticipated to influence the job market and service industries, though the scope and nature of this impact are subject to ongoing research and discussion. While there is potential for AI-driven automation to affect traditional job roles, generative AI also presents opportunities for creating new job positions and services. These emerging roles and services, indicative of the evolving nature of the AI-driven economic landscape, could contribute to new areas of economic growth and innovation. Its integration into various sectors is likely to result in cost efficiencies, operational improvements, and the emergence of new job roles and services, collectively contributing to a global economic transformation in the AI era. The continual developments in this field highlight the importance of ongoing research and analysis to fully understand and capitalize on the economic potential of generative AI.

### 4.8 Challenges and opportunities

Generative AI, while groundbreaking, presents new ethical problems. We must consider how to effectively address the emergence of AI-generated fake news or deepfakes. Moreover, it is crucial to ensure these technologies are used equitably and do not reinforce existing social biases. Yet, these issues also open doors to new governance models, the ethical design of AI, and meaningful public discussions about the future we aspire to create with these technologies.

Security vulnerabilities are a significant concern in generative AI. A key issue is the susceptibility of AI models to manipulation, which could significantly compromise the effectiveness of systems like spam filters and pose potential security risks.

**Figure 8:**
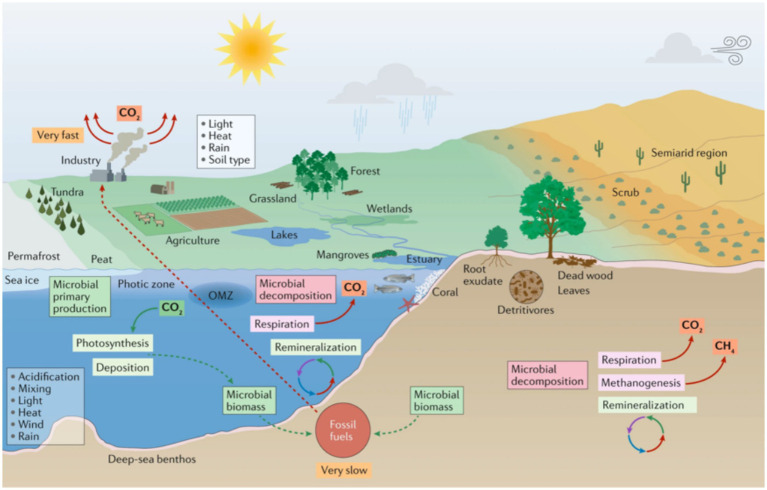
Microorganisms and climate change in marine and terrestrial biomes (Cavicchioli et al., 2019).

**Figure 9:**
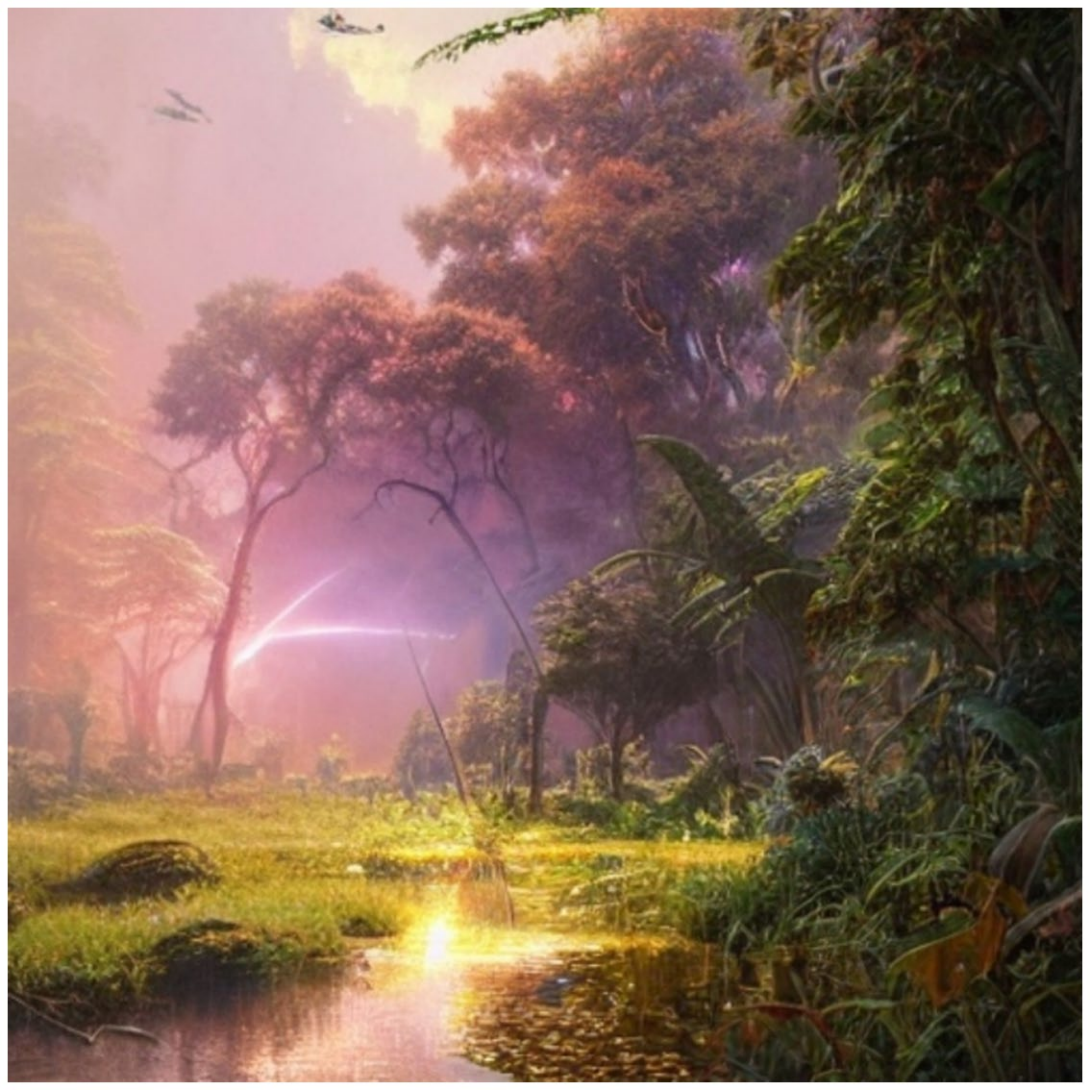
Climate impact of generative AI—deepai.org.

Another pressing issue is the misuse of deepfake technology. This technology’s potential for spreading misinformation, as seen in various contexts, underscores the need for robust security measures to mitigate these risks. While deepfake technology was initially used only for face replacement, with its advancement, misinformation can spread in various areas, such as climate change. In this case, images can easily be manipulated to misrepresent reality, see the image in Figure 8 of nature paper (Cavicchioli et al., 2019), and alternative image generated by Generative AI in Figure 9.

Additionally, the sophistication of AI-generated phishing emails represents an evolving challenge in cybersecurity. This development necessitates advancing defensive strategies to protect against such automated cyber threats.

The use of generative AI has raised concerns regarding security vulnerabilities. One such vulnerability is the susceptibility of AI models to manipulation, as demonstrated by Biggio et al. (2012). They showed that spam filter performance could be severely compromised due to malicious inputs, serving as a warning for potential security breaches in AI systems.

Another significant concern is the potential misuse of deepfake technology, as highlighted by Korshunov and Marcel (2018). This has significant implications for spreading misinformation. The 2020 US election deepfake incidents further emphasize the need for robust security measures.

As Roberts et al. (2021) demonstrated, the sophistication of AI-generated phishing emails represents a new frontier in cybersecurity threats. This underscores the need for advanced defensive strategies to protect against automated cyber-attacks.

## 5 Discriminative vs. generative models

Discriminative models act like judges, with the main task of differentiating or classifying different types of data. For example, if you have a basket of fruits and you want to separate apples from oranges, a discriminative model will learn the boundary that distinguishes the two.

On the other hand, generative models act like artists. They are not concerned with separating apples from oranges. Instead, they can create or generate new fruit similar to what it has seen during training. So, if a generative model is trained on apples and oranges, it has the potential to generate a new variety of apples or oranges.

There are key differences between these two models. Discriminative models give you a label, such as “This is an apple,” while generative models create new data, such as “Here’s a new kind of apple.” Discriminative models learn the boundaries between classes, while generative models learn the distribution of a single data class.

Discriminative models are primarily used for tasks like classification, while generative models have a broader range of applications, including data generation, text completion, and much more.

## 6 Analysis of generative vs. discriminative AI

Artificial intelligence has led to developing two main machine learning models: generative and non-generative (also known as discriminative) models. Both models have unique characteristics that make them suitable for different tasks. Non-generative models are best suited for data classification tasks and are generally easier to train. On the other hand, generative models offer more capabilities, such as data generation and semi-supervised learning, but require more computational resources and may be subject to biases. It is important to understand these models to make informed decisions about which model to use for a particular project, leading to more effective and efficient solutions.

Non-generative or discriminative models are designed to distinguish between different categories or classes. As the name suggests, these models aim to identify the decision boundary that separates distinct categories. For example, Support Vector Machines (SVM) identify hyperplanes that best separate data into distinct classes (Platt, 1999).

In classification tasks, non-generative models, such as logistic regression, are commonly used to recognize benign and malignant tumors in medical diagnostics (Chhatwal et al., 2009). Convolutional Neural Networks (CNNs) excel at identifying and classifying objects within images for image recognition (Krizhevsky et al., 2012).

Generative models are designed to identify and replicate the data distribution of their training sets, unlike discriminative models that aim to classify the input data. These models can generate new instances that closely resemble the original data by capturing the inherent patterns and variations within the data.

Generative Adversarial Networks (GANs) are widely used to create realistic images and art. Goodfellow et al. (2014), but autoencoders have demonstrated significant efficacy in data denoising, enabling audio restoration applications (Lu et al., 2013). Moreover, generative models have proven useful in natural language processing; for instance, language models such as GPT-2 (Radford et al., 2019) can generate text that is frequently indistinguishable from human-generated content.

### 6.1 Comparative analysis

In machine learning, there are two main models: generative and non-generative. Non-generative models are designed to learn the boundaries that separate different classes, which makes them optimal for categorization tasks. On the other hand, generative models aim to capture the underlying data distribution, enabling them to create new data instances.

Regarding capabilities, non-generative models are specialized for classification and regression tasks but lack the inherent ability to produce new data. On the other hand, generative models can generate new data instances and are also helpful in semi-supervised learning scenarios where labelled data is scarce (Kingma et al., 2014).

Non-generative models are limited to the classes they were trained on and require less computational power, while generative models are computationally expensive and require larger training datasets (Goodfellow et al., 2014).

The preceding section provided a thorough academic overview of the distinctions between generative and non-generative models. This was supported by robust empirical studies and specific examples, making it crucial for AI practitioners and researchers to understand these differences clearly. The selection of which model to use is highly dependent on a project’s specific requirements, so a strong grasp of these distinctions is essential for confidently navigating AI development.

The key differences are outlined in Table 1.

**Table 1:** Comparison table of the key differences between generative and discriminative AI.

Criteria	Non-generative models	Generative models
Learning Approach	Learn to differentiate	Learn to generate
Capabilities	Classification, regression	Data generation, semi-supervised learning
Common algorithms	SVM, logistic regression	GANs, autoencoders
Use-cases	Spam filters, image recognition	Art generation, data augmentation
Limitations	Limited to existing classes	May require more data, susceptible to biases

Exploring generative and non-generative models in AI provides invaluable insights into their distinct capabilities and limitations. Non-generative models excel in classification and regression tasks, leveraging their ability to discern and categorize different data classes. In contrast, generative models can generate new data instances and are pivotal in art creation, data augmentation, and semi-supervised learning. The choice between these models hinges on the specific requirements of a project. For tasks requiring precise classification, non-generative models are more suitable, whereas, for projects that benefit from the creation of new data or dealing with limited labelled data, generative models are advantageous. This comparative analysis, encapsulated in Table 1, is essential for AI practitioners and researchers. It guides them in selecting the most appropriate model for their unique objectives, thereby optimizing the efficacy and innovation potential of their AI-driven projects.

## 7 Core technologies behind generative AI

Neural networks are at the core of modern AI and are used in many generative models. They are designed to mimic the neural networks in the human brain, allowing machines to learn from data. Neural networks comprise layers of interconnected nodes or “neurons,” where the output of one layer serves as the input for the next. Convolutional neural networks (CNNs) are commonly used in image recognition tasks.

Autoencoders are neural networks that learn to compress and reconstruct input data. They consist of two parts: an encoder that compresses the input data into a lower-dimensional representation and a decoder that reconstructs the original input from the lower-dimensional representation. Autoencoders are helpful for tasks such as image denoising and dimensionality reduction.

Generative Adversarial Networks (GANs) are generative models that learn to generate new data similar to a given dataset. GANs consist of two neural networks: a generator that generates new data and a discriminator that tries to distinguish between generated and real data. The generator learns to generate better data by trying to fool the discriminator, while the discriminator learns to distinguish between real and generated data.

Transformer Models are neural network architectures for natural language processing tasks such as translation and text generation. They use a self-attention mechanism to process input data and generate output. The most well-known transformer model is the GPT (Generative Pre-trained Transformer) series, with the latest GPT-3. GPT-4 is currently in development.

Neural networks are the foundation of modern AI and are widely used in generative models. Autoencoders learn to compress and reconstruct input data, while GANs learn to generate new data similar to a given dataset. Transformer models, such as GPT-4, are based on a self-attention mechanism for natural language processing tasks (Krizhevsky et al., 2012). The CNN layers’ capability to capture spatial hierarchies makes them an excellent precursor for image-generating models. Neural networks frequently adopt more complex architectures when transitioning to generative paradigms to adequately model complex data distributions.

Autoencoders are a type of neural network specifically designed for unsupervised learning tasks. They consist of two primary components: the encoder and the decoder. The encoder compresses the input data into a lower-dimensional latent space, and the decoder reconstructs the data from this latent representation. Autoencoders have been used in numerous applications, such as dimensionality reduction, anomaly detection, and, notably, in generative tasks (Hinton and Salakhutdinov, 2006). For instance, Variational Autoencoders (VAEs) provide a probabilistic method for describing observations, thereby capturing the inherent uncertainties associated with data generation (Kingma et al., 2014). In practice, VAEs are frequently utilized to generate similar new data to the training data, such as synthesizing new molecules for drug discovery.

Goodfellow et al. (2014) introduced Generative Adversarial Networks (GANs) in 2014, making them one of the most well-known generative models. A Generative Adversarial Network (GAN) comprises two neural networks: the generator and the discriminator. These networks are trained simultaneously in a game of cat and mouse. The generator aims to create indistinguishable data from real data, while the discriminator seeks to differentiate between genuine and artificially generated data. GANs have a broad range of applications, including generating artwork that has been sold for substantial amounts at auction houses like Christie’s (Elgammal et al., 2017) and generating realistic medical imaging data for research (Dar et al., 2019). These models can generate high-quality data, often to the point where it is difficult to distinguish them from actual data.

Transformer models, originating from the natural language processing (NLP) field, have taken generative tasks to an unparalleled level. Initially designed for machine translation, Transformer architecture has evolved into models like GPT (Generative Pre-trained Transformer). GPT-4 is a state-of-the-art example of Transformer-based generative models (Brown et al., 2020). GPT-4 is an advanced artificial intelligence technology that can generate text that makes sense and is relevant to the context. Thanks to its complex neural architecture, it also has some basic comprehension and problem-solving abilities. Its potential diverse applications include automated customer service, content creation, and even scientific research assistance by generating hypotheses or writing code.

## 8 Use cases and applications

In Art and Design, Generative AI offers many new opportunities, from automated design layouts to the creation of intricate artworks. One such platform is “Artbreeder” which allows artists to explore and create new works by combining different elements and styles. Data Augmentation is another area where AI is making a significant impact, allowing for the creation of diverse and larger datasets, which can improve the accuracy and robustness of machine learning models. Text Generation and NLP, or Natural Language Processing, are other areas where AI is used to create more human-like responses and generate coherent and engaging text. In Virtual Reality and Simulations, AI creates more immersive experiences, allowing users to interact with virtual environments in new and exciting ways. Finally, in the Breakout Room Discussion, participants will explore and imagine the future applications of AI in various fields [23]. For instance, Generative Adversarial Networks (GANs) can merge different images or art styles, allowing users to create unique and original works of art. Additionally, Artificial Intelligence (AI) systems can generate architectural designs, allowing architects to explore unconventional and computationally complex structures. Using Generative AI techniques, the architectural firm Zaha Hadid Architects proposes avant-garde building designs that push the boundaries of traditional aesthetics and functionality (Zaha Hadid Architects, 2023).

Data Augmentation is an important application of Generative AI. GANs have been used to augment existing medical image datasets to enhance diagnostic algorithms’ effectiveness in medical research. Frid-Adar et al. (2018) demonstrated that GANs can generate synthetic Computed Tomography (CT) images, which, when combined with actual CT scans, significantly improved the performance of lung nodule classification models. This data augmentation capability addresses the limitations of small or unbalanced datasets and has profound implications for fields inherently constrained by data availability.

Text Generation and Natural Language Processing (NLP) have shown great potential with Generative AI. OpenAI’s GPT-4 model has set new language comprehension and generation benchmarks. These models can produce logically coherent and contextually relevant text over long passages, making them invaluable for automated content creation, summarization, and machine translation. One notable application of text generation is the creation of synthetic yet realistic legal contracts for preliminary reviews, significantly saving time and effort. However, there is a need for further research into the ethical aspects of text generation, particularly in misinformation and content authenticity.

Generative AI has wide-ranging applications in the fields of virtual reality and simulations. For example, NVIDIA has developed deep learning-based image synthesis techniques to generate highly realistic virtual training environments for autonomous vehicles. These simulations cover various driving conditions and scenarios, providing a comprehensive training framework. Furthermore, the airline industry is exploring the potential of Generative AI to develop more realistic flight simulators for pilot training. As these simulations become increasingly similar to real-world situations, the effectiveness of the training programs increases exponentially.

## 9 Limitations of generative AI and ethical considerations

The development of AI models faces certain limitations and challenges. One of the key challenges is the high computational cost associated with the training and generation phases. Creating a convincing deepfake requires a large dataset and significant computational power. However, this resource-intensive nature of AI not only restricts accessibility but also raises environmental concerns due to the energy consumption of the data centers that run these models.

Another limitation is that AI models heavily rely on the quality of the training data. Thus, the quality of the generated output is only as good as the quality of the training data. Therefore, if the data used to train the AI model is biased or misleading, the AI model can perpetuate and amplify those biases, negatively affecting the accuracy and fairness of the generated content. This is particularly important in cases where ethical dilemmas such as deepfakes are involved.

The rapid development of technology has brought about many advancements that have significantly improved our lives. However, some of these advancements have also raised significant concerns. One such concern is the development of technologies that allow video and audio manipulation with an extreme degree of authenticity.

The most well-known of these technologies are deepfakes, which are synthetic media that can show people doing or saying things they never actually did. The ability of deepfakes to generate synthetic media that is difficult to distinguish from the real thing raises serious concerns about identity theft and invasion of privacy. Such deepfakes can cause significant personal, professional, and reputational harm. For example, a CEO’s deepfake speech in a fake announcement caused a company’s stock to plummet, resulting in financial losses.

Moreover, deepfakes also significantly threaten the veracity of news and information. In a politically charged instance, a deepfake video purporting to show a politician engaging in corrupt practices was distributed. Even after the video was debunked, public confidence and the damage to the electoral process were irreparable.

The legal implications of deepfakes are also significant. Current laws are inadequate to address the problems posed by deepfakes. While defamation laws may protect victims, they still bear the burden of proving falsity and malice. The ease with which deepfakes can cross international borders exacerbates the legal complexities.

The development of technologies that allow video and audio manipulation with an extreme degree of authenticity has raised serious concerns about identity theft, invasions of privacy, the veracity of news and information, and the legal implications of deepfakes. Addressing these concerns requires developing new technologies that can detect deepfakes and improving our laws to better protect victims of deepfakes.

## 10 Integrated theoretical framework for generative AI governance

Building on the thematic insights identified in our systematic literature review (section 3), this section presents an integrated theoretical framework designed to address the ethical, security, and privacy challenges posed by generative AI systems. The framework is informed by established theories in technology adoption, cybersecurity, and ethical governance, and synthesizes conceptual elements drawn from empirical findings and normative guidelines discussed earlier in this paper.

At the core of the framework is a three-tiered structure aligned with the PRISMA-derived thematic clusters: (1) *Adoption and Acceptance*, (2) *Security and Resilience*, and (3) *Ethical and Regulatory Alignment*. The first tier incorporates established adoption models, most notably the *Diffusion of Innovations Theory* (Rogers) and the *Technology Acceptance Model* (TAM), to model how generative AI systems gain traction within different institutional contexts. This includes user perception of utility, system usability, and the role of social norms in shaping AI adoption behaviors. These models are foundational in capturing the socio-technical dynamics that influence early adoption, resistance, or rejection of generative systems, particularly in sectors such as healthcare and finance.

The second-tier addresses cybersecurity imperatives and is underpinned by the *CIA Triad* (Confidentiality, Integrity, and Availability) as well as the NIST Cybersecurity Framework (NIST Cybersecurity, 2016). These principles provide a normative scaffold for defining resilience in AI systems against adversarial threats such as model poisoning, data exfiltration, and automated misinformation. The inclusion of adversarial training, model robustness testing, and threat modelling supports proactive risk mitigation, directly responding to the vulnerabilities highlighted in Section 6 and our quantitative results.

The third-tier addresses ethics and governance by incorporating normative principles from the *Asilomar AI Principles*, *IEEE’s Ethically Aligned Design*, and *GDPR-compliant privacy regimes*. These components collectively ensure that AI development respects human dignity, ensures accountability, and maintains proportionality in data usage. The framework operationalizes these norms by proposing implementation tools such as algorithmic auditing, explainability-by-design, consent management, and differential privacy—all of which are grounded in the use cases and privacy risks explored in Sections 7 and 8.

**Figure 10:**
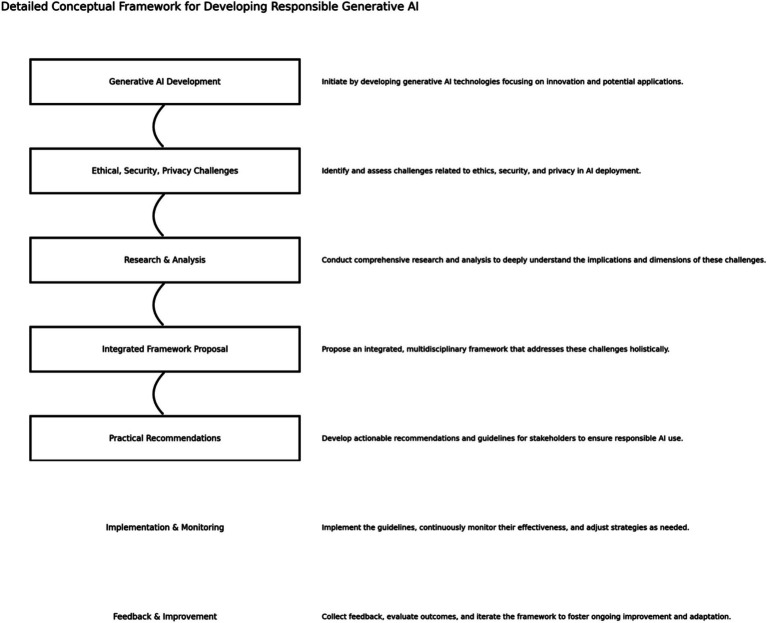
Comprehensive guide for practitioners, outlining a clear path from the development of AI technologies to the responsible implementation and continuous improvement of AI systems.

Figure 10 illustrates the framework as a modular and iterative pipeline, from design and deployment through to monitoring and governance, thereby enabling practitioners to evaluate generative AI systems through the lenses of usability, security posture, and ethical conformity. Unlike traditional risk management models, our framework offers a cyclical structure that integrates continuous feedback and self-correction, supporting resilience over time.

In doing so, the framework moves beyond theoretical abstraction and delivers a practical schema for developers, regulators, and end-users. By embedding it within a multi-layered structure that reflects the reviewed literature and empirical findings, the framework directly addresses the reviewer’s call for conceptual coherence and methodological justification.

The framework in Figure 10 collectively provides the basis for understanding and addressing the challenges of adopting generative AI while ensuring security, ethical integrity, and privacy protection. It guides the development and implementation of generative AI in a socially responsible, ethically sound manner and in compliance with established norms and regulations.

The proposed theoretical framework synthesized in Table 2 offers a structured, multi-dimensional approach to responsible generative AI deployment. Grounded in established theories and regulatory standards, the framework integrates perspectives from technology adoption, cybersecurity resilience, and ethical governance. It is organized across five sequential lifecycle stages, ranging from system design through to post-deployment feedback, and maps these against three foundational tiers: user adoption and acceptance, technical security and resilience, and regulatory and ethical alignment. This structure allows practitioners and researchers to operationalize complex theoretical insights within real-world AI system lifecycles, ensuring both robustness and accountability in generative AI applications.

**Table 2:** Integrated framework for responsible generative AI deployment.

Lifecycle stage	Tier 1: adoption and acceptance	Tier 2: security and resilience	Tier 3: ethics and regulation
1. System design and objectives	- Define user needs and expectations—Map stakeholders—Anticipate adoption barriers	- Apply CIA Triad in architecture design—Identify attack surfaces—Embed secure coding practices	- Conduct Data Protection Impact Assessments (DPIA)—Map ethical risks—Apply principles from Asilomar & IEEE
2. Implementation and adoption	- Ensure usability and accessibility—Align with TAM constructs (usefulness, ease-of-use)	- Implement adversarial training—Use sandbox testing for vulnerabilities	- Integrate privacy-by-design—Review for bias/fairness—Apply GDPR data handling constraints
3. Monitoring and risk assessment	- Collect adoption metrics—Evaluate user satisfaction—Observe behavioral adaptation	- Conduct penetration testing—Monitor for adversarial inputs—Validate model robustness	- Perform algorithmic audits—Check for transparency & explainability gaps—Ensure ongoing consent
4. Policy and compliance alignment	- Align with organizational digital policy—Embed AI guidelines into internal culture	- Apply NIST Cybersecurity Framework—Ensure system auditability and logging	- Align with GDPR, HIPAA, sectoral laws—Use FIPPs for data governance—Adopt AI Act/ISO AI standards
5. Feedback and recalibration	- Gather end-user feedback for retraining—Update UI/UX based on engagement data	- Patch known exploits—Use red-teaming and stress tests—Re-tune resilience metrics	- Update compliance documents—Re-audit models post-deployment—Reassess fairness and accountability

Table 2 explains how each lifecycle phase incorporates distinct, yet interdependent, responsibilities across the three tiers. In the early design phase, emphasis is placed on identifying user needs, embedding security architectures such as the CIA Triad, and anticipating legal and ethical implications via instruments like data protection impact assessments. As systems move into implementation and deployment, the framework calls for usability testing, adversarial robustness methods, and privacy-by-design protocols. During monitoring, it encourages both quantitative (e.g., threat modelling, stress testing) and qualitative (e.g., user trust evaluation, transparency audits) assessments. Policy alignment is achieved through compliance with domain-specific standards such as GDPR, NIST, and IEEE. Finally, the feedback and recalibration phase ensures that AI systems remain adaptive, ethical, and resilient through continual learning, stakeholder engagement, and re-certification. This lifecycle-integrated perspective ensures the framework is both theoretically grounded and practically actionable.

## 11 Discussion: operationalizing resilience in generative AI deployment

This study has highlighted the dual potential of generative AI to drive innovation and simultaneously introduce critical risks related to security, privacy, and ethical integrity. The findings presented throughout the paper, particularly the PRISMA-guided literature review, the empirical case analysis, and the integrated theoretical framework, demonstrate that the responsible deployment of generative AI cannot be approached as a purely technical endeavor. Instead, it must be understood as a socio-technical challenge requiring layered governance, stakeholder alignment, and adaptive security mechanisms.

The integrated framework introduced in section 9 provides a practical blueprint for stakeholders to address these complexities across the entire AI lifecycle. For instance, early-stage design choices must not only consider model efficiency and computational optimization but also pre-empt usability and fairness, as identified in the adoption and acceptance tier. This is particularly relevant in sectors such as healthcare, where trust in AI-generated diagnostics depends on perceived utility and transparency. The implementation phase must similarly be informed by adversarial training and sandbox testing, as discussed in the cybersecurity tier, to mitigate threats such as model poisoning or deepfake synthesis, risks identified in both the empirical and bibliometric analyses.

Moreover, our results demonstrate that monitoring and risk assessment are not static procedures but must evolve through continuous threat modelling, algorithmic audits, and engagement with compliance standards such as GDPR, NIST, and ISO AI frameworks. These findings validate the policy alignment tier of the framework and point toward the growing convergence of technical standards and ethical mandates. For instance, privacy-preserving techniques like federated learning and differential privacy, when applied proactively, serve not only as protective mechanisms but also as compliance enablers.

Through thematic synthesis, we also identified gaps between emerging use cases, such as generative AI in creative production, diagnostics, or climate modelling, and current governance regimes. These use cases illustrate the urgency of translating abstract ethical principles into enforceable protocols, as shown in the ethical and regulatory alignment tier. Real-world scenarios, such as the re-identification risks in anonymized health datasets and the spread of synthetic misinformation via deepfakes, underline the need for integrated policy responses that combine technical vigilance with regulatory agility.

Ultimately, resilience in generative AI must be understood as a dynamic and cross-disciplinary construct, and sustaining accountability, trust, and adaptability in rapidly evolving socio-technical systems.

## 12 Conclusion

This study has critically examined the security, ethical, and privacy implications of generative AI technologies and proposed a multi-layered governance framework to enhance their resilience across domains such as healthcare, cybersecurity, and creative industries. Drawing on a systematic literature review guided by the PRISMA framework, combined with qualitative thematic analysis and quantitative evaluation, this research has identified persistent gaps in the integration of governance mechanisms, socio-technical resilience, and regulatory compliance in current AI deployments.

A key contribution of this work is the development of an *Integrated Framework for Responsible Generative AI Deployment*, which maps governance strategies across the AI lifecycle, from system design to post-deployment recalibration. The framework operationalizes theoretical constructs from technology adoption (e.g., TAM, Diffusion of Innovations), cybersecurity (e.g., CIA Triad, NIST), and ethical governance (e.g., GDPR, IEEE, Asilomar Principles), offering a unified, actionable model for responsible deployment. Through the introduction of this framework, the study provides both a conceptual lens and a practical roadmap for AI developers, regulators, and institutional adopters seeking to embed trust, accountability, and robustness into generative AI systems.

The findings of this study reveal that while generative AI enables transformative capabilities (from synthetic data generation to multimodal content creation) it simultaneously introduces risks such as adversarial manipulation, re-identification of anonymized data, and deepfake proliferation. These risks are amplified by the rapid diffusion of generative models in sectors that lack mature governance ecosystems. As such, resilience must be redefined in technical terms but also in terms of ethical accountability and policy adaptability.

This work contributes to the academic discourse by bridging the often-disconnected conversations between AI engineering, digital ethics, and regulatory studies. It advances a holistic perspective that acknowledges the socio-technical complexity of deploying generative AI at scale. The framework presented is intended to be used as a dynamic tool that can evolve with the technological and regulatory landscape.

Looking ahead, future research should focus on empirically validating the framework across specific sectors through longitudinal case studies and stakeholder-driven evaluation. Further work is also needed to quantify resilience metrics in generative AI systems and to integrate real-time threat detection, ethical auditing, and user feedback mechanisms into scalable AI infrastructures. By doing so, we can ensure that generative AI development proceeds with technical ambition, ethical foresight, and social responsibility.

## References

[ref1] Acemoglu D. Restrepo P. (2019). The wrong kind of AI? Artificial intelligence and the future of labor demand. in NBER working paper series, Cambridge, MA: National Bureau of Economic Research. doi: 10.3386/W25682

[ref2] African Union. African union convention on cyber security and personal data protection|African Union. Available online at: https://au.int/en/treaties/african-union-convention-cyber-security-and-personal-data-protection (2020) (Accessed July 25, 2023).

[ref3] Antoniou A. Storkey A. Edwards H. (2018). Augmenting image classifiers using data augmentation generative adversarial networks. Lecture Notes Comput. Sci. 11141, 594–603. doi: 10.1007/978-3-030-01424-7_58, PMID: 40331156

[ref4] Bartoletti I. AI in healthcare: ethical and privacy challenges. Lecture notes in computer science (including subseries lecture notes in artificial intelligence and lecture notes in bioinformatics). Springer Nature Link. (2019).

[ref5] Beers A. Brown J. Ken C. Peter Campbell J. Ostmo S. Chiang M. F.. High-resolution medical image synthesis using progressively grown generative adversarial networks. Available online at: https://arxiv.org/abs/1805.03144v2 (2018) (Accessed September 16, 2024).

[ref6] Biggio B. Nelson B. Laskov P. (2012). Poisoning attacks against support vector machines. Proceedings of the 29th international conference on machine learning. ICML 2, 1807–1814. doi: 10.48550/arXiv.1206.6389

[ref7] Brown T. B. Mann B. Ryder N. Subbiah M. Kaplan J. Dhariwal P.. Language models are few-shot learners. Advances in Neural Information Processing Systems. (2020). Available at: https://arxiv.org/abs/2005.14165v4 (Accessed December 27, 2023).

[ref8] Buolamwini J. Gebru T. (2018). Gender shades: intersectional accuracy disparities in commercial gender classification. Proc. Mach. Learn. Res. 81, 77–91. Available at: https://proceedings.mlr.press/v81/buolamwini18a/buolamwini18a.pdf

[ref9] Buzuti L. F. Thomaz C. E. (2023). Fréchet auto encoder distance: a new approach for evaluation of generative adversarial Networks. Comput. Vis. Image Underst. 235:103768. doi: 10.1016/j.cviu.2023.103768

[ref10] Carlini N. Wagner D.. MagNet and ‘efficient defenses against adversarial attacks’ are not robust to adversarial examples, Available online at: http://arxiv.org/abs/1711.08478 (2017) (Accessed November 15, 2024).

[ref11] Carole C. Emma G.-H. (2018). Revealed: 50 million Facebook profiles harvested for Cambridge Analytica in major data breach. Guardian. Available at: https://www.theguardian.com/news/2018/mar/17/cambridge-analytica-facebook-influence-us-election (Accessed May 16, 2025).

[ref12] Cavicchioli R. Ripple W. J. Timmis K. N. Azam F. Bakken L. R. Baylis M.. (2019). Scientists’ warning to humanity: microorganisms and climate change. Nat. Rev. Microbiol. 17, 569–586. doi: 10.1038/s41579-019-0222-5 31213707 PMC7136171

[ref13] Chen S. Carlini N. Wagner D. (2020). Stateful detection of black-box adversarial attacks. SPAI 2020”- proceedings of the 1st ACM workshop on security and privacy on arti!cial intelligent. Co-locat Asia CCS 2020, 30–39. doi: 10.1145/3385003.3410925

[ref15] Chhatwal J. Alagoz O. Lindstrom M. J. Kahn C. E. Jr Shaffer K. A. Burnside E. S. (2009). A logistic regression model based on the national mammography database format to aid breast cancer diagnosis. Am. J. Roentgenol. 192, 1117–1127. doi: 10.2214/AJR.07.3345, PMID: 19304723 PMC2661033

[ref16] CVE. CVE security vulnerability database. Security vulnerabilities, exploits, references and more. Available at: https://www.cvedetails.com/ (2022) (Accessed January 3, 2023).

[ref18] Dar S. U. H. Yurt M. Karacan L. Erdem A. Erdem E. Cukur T. (2019). Image synthesis in multi-contrast MRI with conditional generative adversarial Networks. IEEE Trans. Med. Imaging 38, 2375–2388. doi: 10.1109/TMI.2019.2901750, PMID: 30835216

[ref19] Deng Z. Guo Y. Han C. Ma W. Xiong J. Wen S.. AI agents under threat: a survey of key security challenges and future pathways. 1. Available online at: https://arxiv.org/abs/2406.02630v2 (2024) (Accessed September 12, 2024).

[ref20] Ding X. Wang Y. Xu Z. Welch W. J. Wang Z. J. (2021). “Ccgan: continuous conditional generative adversarial networks for image generation” in International Conference on Learning Representations.

[ref21] Du X. Zhang Q. Zhu J. Liu X. (2024). Adaptive unified defense framework for tackling adversarial audio attacks. Artif. Intell. Rev. 57, 1–22. doi: 10.1007/s10462-024-10863-7, PMID: 40332530

[ref22] Elgammal A. Liu B. Elhoseiny M. Mazzone M. (2017). CAN: creative adversarial networks, generating ‘art’ by learning about styles and deviating from style norms. Proceedings of the 8th International Conference on Computational Creativity, ICCC 2017. Available at: https://arxiv.org/abs/1706.07068v1 (Accessed September 4, 2023).

[ref23] Esteve A. (2017). The business of personal data: Google, Facebook, and privacy issues in the EU and the USA. Int. Data Privacy Law 7, 36–47. doi: 10.1093/idpl/ipw026

[ref24] European Commission. Ethics guidelines for trustworthy AI|Shaping Europe’s digital future. Available online at: https://digital-strategy.ec.europa.eu/en/library/ethics-guidelines-trustworthy-ai (2018) (Accessed July 7, 2023).

[ref25] Frid-Adar M. Diamant I. Klang E. Amitai M. Goldberger J. Greenspan H. (2018). GAN-based synthetic medical image augmentation for increased CNN performance in liver lesion classification. Neurocomputing 321, 321–331. doi: 10.1016/j.neucom.2018.09.013

[ref26] GDPR. What is GDPR, the EU’s new data protection law? GDPR.eu. Available online at” https://gdpr.eu/what-is-gdpr/ (2018). (Accessed July 7, 2023).

[ref27] Gifford G. McCutcheon R. McGuire P. (2020). Neuroimaging studies in people at clinical high risk for psychosis. Risk Fact. Psychos. 2020, 167–182. doi: 10.1016/B978-0-12-813201-2.00009-0

[ref28] Goodfellow I. Pouget-Abadie J. Mirza M. Xu B. Warde-Farley D. Ozair S.. (2014). Generative adversarial networks. Commun. ACM 63, 139–144. doi: 10.1145/3422622, PMID: 39076787

[ref29] Guy Z. PAlex N. O. (2015). “Decentralizing privacy: using blockchain to protect personal data” in Proceedings −2015 IEEE security and privacy workshops, SPW (Institute of Electrical and Electronics Engineers Inc), 180–184.

[ref30] He Y. Wang E. Rong Y. Cheng Z. Chen H. (2024). Security of AI Agents. Available online at: https://arxiv.org/abs/2406.08689v2 (Accessed August 29, 2024).

[ref31] Hinton G. E. Salakhutdinov R. R. (2006). Reducing the dimensionality of data with neural networks. Science 313, 504–507. doi: 10.1126/science.1127647 16873662

[ref32] Hosny A. Parmar C. Quackenbush J. Schwartz L. H. Aerts H. J. W. L. (2018). Artificial intelligence in radiology. Nat. Rev. Cancer 18, 500–510. doi: 10.1038/s41568-018-0016-5, PMID: 29777175 PMC6268174

[ref33] ICO. Information Commissioner’s Office (ICO): THE UK GDPR. UK GDPR guidance and resources. Available online at: https://ico.org.uk/for-organisations/data-protection-and-the-eu/data-protection-and-the-eu-in-detail/the-uk-gdpr/ (2018) (Accessed July 8, 2023).

[ref34] IEEE. IEEE introduces new program for free access to AI ethics and governance standards (2023). Available at: https://standards.ieee.org/news/get-program-ai-ethics/

[ref35] Jobin A. Ienca M. Vayena E. (2019). The global landscape of AI ethics guidelines. Nat Mach Intell 1, 389–399. doi: 10.1038/s42256-019-0088-2

[ref36] Karras T. Laine S. Aila T.. A style-based generator architecture for generative adversarial networks. Proceedings of the IEEE Computer Society Conference on Computer Vision and Pattern Recognition (2019) 4396–4405. doi: 10.1109/CVPR.2019.00453

[ref37] Kendzierskyj S. Jahankhani H. Hussien O. A. A. M. (2024). Space governance frameworks and the role of AI and quantum computing, Space Law and Policy (SLP), Springer Nature. 1–39. doi: 10.1007/978-3-031-62228-1_1, PMID: 40331156

[ref38] Kenfack P. J. Arapov D. D. Hussain R. Ahsan Kazmi S. M. Khan A. On the fairness of generative adversarial networks (GANs), 2021 International Conference ‘Nonlinearity, Information and Robotics’, NIR (2021). doi: 10.1109/NIR52917.2021.9666131

[ref39] Khamaiseh S. Y. Bagagem D. Al-Alaj A. Mancino M. Alomari H. W. (2022). Adversarial deep learning: a survey on adversarial attacks and defense mechanisms on image classification. IEEE Access 10, 102266–102291. doi: 10.1109/ACCESS.2022.3208131

[ref40] Kim E. Cho H. H. Cho S. H. Park B. Hong J. Shin K. M.. (2022). Accelerated synthetic MRI with deep learning-based reconstruction for pediatric neuroimaging. AJNR Am. J. Neuroradiol. 43, 1653–1659. doi: 10.3174/ajnr.A7664, PMID: 36175085 PMC9731246

[ref41] Kingma D. P. Rezende D. J. Mohamed S. Welling M. (2014). Semi-supervised learning with deep generative models. Adv Neural Inf Process Syst 27, 1–9. doi: 10.48550/arXiv.1406.5298

[ref42] Korshunov P. Marcel S.. DeepFakes: a new threat to face recognition? Assessment and detection, Available online at: https://arxiv.org/abs/1812.08685v1 (2018) (Accessed December 27, 2023).

[ref43] Kostka G. (2019). China’s social credit systems and public opinion: explaining high levels of approval. New Media Soc. 21, 1565–1593. doi: 10.1177/1461444819826402

[ref44] Krizhevsky A. Sutskever I. Hinton G. E. ImageNet classification with deep convolutional neural networks. (2012) Available online at: http://code.google.com/p/cuda-convnet/ (Accessed September 3, 2023).

[ref45] Kutuzova S. Krause O. McCloskey D. Nielsen M. Igel C. Multimodal Variational autoencoders for semi-supervised learning: In Defense of product-of-experts. Available online at: https://arxiv.org/abs/2101.07240v2 (2021) (Accessed September 19. 2024).

[ref46] Lawry Aguila A. Chapman J. Altmann A. (2023). Multi-modal variational autoencoders for normative modelling across multiple imaging modalities. Lect. Notes Comput. Sci. 14220, 425–434. doi: 10.1007/978-3-031-43907-0_41

[ref47] Lu X. Tsao Y. Matsuda S. Hori C. Speech enhancement based on deep denoising autoencoder. Proceedings of the Annual Conference of the International Speech Communication Association, INTERSPEECH (2013); 436–440. doi: 10.21437/INTERSPEECH.2013-130

[ref48] Miaoui Y. Boudriga N. (2019). Enterprise security investment through time when facing different types of vulnerabilities. Inf. Syst. Front. 21, 261–300. doi: 10.1007/s10796-017-9745-3

[ref49] Mishra S. (2023). Exploring the Impact of AI-Based Cyber Security Financial Sector Management. Appl. Sci. 13:5875. doi: 10.3390/APP13105875

[ref50] Mökander J. Schuett J. Kirk H. R. Floridi L. (2024). Auditing large language models: a three-layered approach. AI Ethics 4, 1085–1115. doi: 10.1007/s43681-023-00289-2

[ref51] NIST Cybersecurity. Cybersecurity Framework|NIST, Available online at: https://www.nist.gov/cyberframework (2016).

[ref52] Orphanou K. Otterbacher J. Kleanthous S. Batsuren K. Giunchiglia F. Bogina V.. (2022). Mitigating bias in algorithmic systems - a fish-eye view. ACM Comput Surv 55. doi: 10.1145/3527152/SUPPL_FILE/3527152.SUPP.PDF

[ref53] Platt J. C. Probabilistic outputs for support vector machines and comparisons to regularized likelihood methods. (1999) Available online at: https://www.researchgate.net/publication/2594015 (Accessed September 3, 2023).

[ref54] Porambage P. Kumar T. Liyanage M. Partala J. Lovén L. Ylianttila M.. (2019). Sec-edge AI: AI for edge security vs security for edge AI brain ICU-Measuring brain function during intensive care view project ECG-based emotion recognition view project sec-edge AI: AI for edge security vs security for edge AI. Available online at: https://www.researchgate.net/publication/330838792 (Accessed October 28, 2019).

[ref55] Radford A. Metz L. Chintala S. (2015). Unsupervised representation learning with deep convolutional generative adversarial networks. 4th International Conference on Learning Representations, ICLR 2016- Conference Track Proceedings, Available at: https://arxiv.org/abs/1511.06434v2 (Accessed June 27,2024).

[ref56] Radford A. Jeffrey W. Child R. Luan D. Amodei D. Sutskever I. (2019). Language models are unsupervised multitask learners. OpenAI Blog. Available online at: https://github.com/codelucas/newspaper (Accessed September 3, 2023).

[ref57] Ren K. Zheng T. Qin Z. Liu X. (2020). Adversarial attacks and defenses in deep learning. Engineering 6, 346–360. doi: 10.1016/j.eng.2019.12.012

[ref58] Roberts H. Cowls J. Morley J. Taddeo M. Wang V. Floridi L. (2021). The Chinese approach to artificial intelligence: an analysis of policy, ethics, and regulation. AI Soc. 36, 59–77. doi: 10.1007/s00146-020-00992-2

[ref59] Rocher L. Hendrickx J. M. de Montjoye Y. A. (2019). Estimating the success of re-identifications in incomplete datasets using generative models. Nat. Commun. 10, 1–9. doi: 10.1038/s41467-019-10933-3 31337762 PMC6650473

[ref60] Sandfort V. Yan K. Pickhardt P. J. Summers R. M. (2019). Data augmentation using generative adversarial networks (CycleGAN) to improve generalizability in CT segmentation tasks. Sci. Rep. 9, 1–9. doi: 10.1038/s41598-019-52737-x, PMID: 31729403 PMC6858365

[ref61] Sarker I. H. Furhad M. H. Nowrozy R. (2021). AI-driven cybersecurity: an overview, security intelligence modeling and research directions. SN Comput Sci 2, 1–18. doi: 10.1007/s42979-021-00557-0, PMID: 40332530

[ref62] Sava PA. Schulze JP. Sperl P Böttinger K. Proceedings of the 15th ACM, and undefined 2022. (2024). ‘Assessing the Impact of Transformations on Physical Adversarial Attacks’. doi: 10.1145/3560830.3563733 (Accessed November 15, 2024).

[ref63] Sava P. A. Schulze J. P. Sperl P. Böttinger K. (2022). Assessing the impact of transformations on physical adversarial attacks. AISec 2022- Proceedings of the 15th ACM Workshop on Artificial Intelligence and Security, co-located with CCS 2022. 79–90. doi: 10.1145/3560830.3563733

[ref64] Shi Y. Siddharth N. Paige B. Torr P. H. S. (2019). Variational mixture-of-experts autoencoders for multi-modal deep generative models. Adv Neural Inf Process Syst. 32.

[ref65] Sindhura D. Pai R. M. Bhat S. N.. (2022). “Sub-axial vertebral column fracture CT image synthesis by progressive growing generative adversarial Networks (PGGANs)” in 2022 IEEE International Conference on Distributed Computing, VLSI, Electrical Circuits and ROBOTICS, DISCOVER 2022- Proceedings, 311–315.

[ref17] Silva-Filarder M. Da Ancora A. Filippone M. Michiardi P.. Multimodal Variational autoencoders for sensor fusion and cross generation. Proceedings -20th IEEE international conference on machine learning and applications. ICMLA (2021). 2021; 1069–1076. doi: 10.48550/arXiv.2101.07240

[ref66] Sollee J. Tang L. Igiraneza A. B.. (2022). Artificial intelligence for medical image analysis in epilepsy. Epilepsy Res. 182:106861. doi: 10.1016/j.eplepsyres.2022.106861 35364483

[ref67] Sun L. Tan M. Zhou Z. (2018). A survey of practical adversarial example attacks. Cybersecurity 1, 1–9. doi: 10.1186/s42400-018-0012-9, PMID: 40325206

[ref68] Tedeneke A. World economic forum launches AI governance alliance focused on responsible generative AI. (2023). Available at: https://www.weforum.org/press/2023/06/world-economic-forum-launches-ai-governance-alliance-focused-on-responsible-generative-ai/

[ref69] Wang Q. Luo L. Xie H. Rao Y. Lau R. Y. K. Zhang D. (2022). A deep data augmentation framework based on generative adversarial networks. Multimed. Tools Appl. 81, 42871–42887. doi: 10.1007/s11042-022-13476-w

[ref70] Wang Z. Wallace C. Bifet A. Yao X. Zhang W. (2023). FG2 AN: fairness-aware graph generative adversarial Networks. Lecture Notes Comp. Sci. 14170, 259–275. doi: 10.1007/978-3-031-43415-0_16

[ref71] Wang H. Wu C. Networks K. Z.-N.. (2024). Defense against adversarial attacks based on color space transformation. Neural Netw. 173:106176. doi: 10.1016/j.neunet.2024.106176, PMID: 38402810

[ref72] Welander P. Karlsson S. Eklund A. Generative adversarial networks for image-to-image translation on multi-contrast MR images - a Comparison of CycleGAN and UNIT. Available at: https://arxiv.org/abs/1806.07777v1 (2018) (Accessed September 19, 2024).

[ref73] Wheatley S. Maillart T. Sornette D. (2016). The extreme risk of personal data breaches and the erosion of privacy. Eur. Phys. J. B. 89, 1–12. doi: 10.1140/epjb/e2015-60754-4, PMID: 37198866

[ref74] Zaha Hadid Architects. Zaha Hadid Architects using AI image generators for design concepts, said Patrik Schumacher. Available at: https://parametric-architecture.com/zaha-hadid-architects-using-ai-image-generators-for-design-concepts-said-patrik-schumacher/ (2023) (Accessed September 4, 2023).

[ref75] Zbrzezny A. M. Grzybowski A. E. (2023). Deceptive tricks in artificial intelligence: adversarial attacks in ophthalmology. J. Clin. Med. 12:3266. doi: 10.3390/JCM12093266 37176706 PMC10179065

[ref76] Zhavoronkov A. Ivanenkov Y. A. Aliper A. Veselov M. S. Aladinskiy V. A. Aladinskaya A. V.. (2019). Deep learning enables rapid identification of potent DDR1 kinase inhibitors. Nat. Biotechnol. 37, 1038–1040. doi: 10.1038/s41587-019-0224-x, PMID: 31477924

